# Supplementary material for: ARTEMIS integrates autoencoders and Schrödinger Bridges to predict continuous dynamics of gene expression, cell population, and perturbation from time-series single-cell data
Source: Bioinformatics. 2025 Jul 15;41(Suppl 1):i189–97. doi: 10.1093/bioinformatics/btaf218 (PMC12261462; doi:10.1093/bioinformatics/btaf218)
Supplement: btaf218_Supplementary_Data [file btaf218_supplementary_data.zip › Wang.322.sup.1.pdf]

Supplementary Notes

Algorithm S1 ARTEMIS

Input: $\{X_t\}_{t\in\{0,1,..T\}}, t \in \{0, 1, ..T\}$, prior kill rate k, pretrain epochs E_1, number of iterations E_2, joint train epochs E_3, #SDEs to sample E_4

Initialize: $q_\varphi, p_\phi, Q_\theta, \widehat{Q}_{\widehat{\theta}}, r^\omega$

```
1: Pre-train VAE:
2: for i=1 to E_1 do
3:     q_φ(X_t||t) = (μ_t, σ_t), Z_t ~ N(μ_t, σ_t^2), X̂_t = p_ϕ(Z_t)
4:     Update φ, ϕ using ∇L_vae (Eq. 2)
5: end for
6: Jointly train VAE and uDSB:
7: for j=1 to E_2 do
8:     for k=1 to E_3 do
9:         for l=1 to E_4 do
10:            (←Z_t, A_t) ← sample B-SDE(Q_θ, Q̂_θ̂, K_ω) (Eq. 9b)
11:            Update θ using ∇_θ L_div,θ (Eq. 12b)
12:            sample F-SDE ←Z_t ~ ρ_0 (Eq. 9a)
13:            update θ, (φ, ϕ) using ∇_θ,φ,ϕ L_joint (Eq. 13)
14:        end for
15:        for l=1 to E_4 do
16:            (→Z_t, A_t) ← sample F-SDE(Q_θ, Q̂_θ̂, K_ω)(Eq. 9a)
17:            update θ̂, ω using ∇_θ̂ L_div,θ̂ (Eq. 12a),∇_ω L_ω (Eq. eq:10), respectively
18:        end for
19:    end for
20: end for
21: Outputs:
       φ, ϕ, θ, θ̂, ω
```

S1 Details of model implementation, training, and testing

ARTEMIS is implemented in JAX (Bradbury et al., 2018). Training begins with VAE pre-training on single-cell gene expression data from observed timepoints. The ADAM optimizer with gradient clipping (threshold=1) and initial learning rate as 0.0001 was used and the VAE was trained for E_1 epochs with a default batch size of 64 (Algorithm S1, Steps 1-5).

Subsequently, the uDSB and VAE are jointly trained to learn a smooth latent space. The uDSB model comprises three networks, a.) forward drift Q_θ, b.) backward drift $\widehat{Q}_{\widehat{\theta}}$, and c.) kill rate K_ω.

The architecture of Q_θ and $\widehat{Q}_{\widehat{\theta}}$ includes:

i) `x_encoder`: 3-layer MLP taking Z_t as input, ii) `t_encoder`: 3-layer MLP taking the sinusoidal embedding of time t as input, and, iii) `decoder`: 2-layer MLP decoder combining ouputs from `x_encoder` and `t_encoder` to output optimal drift values (Q_θ,$\widehat{Q}_{\widehat{\theta}}$).

The K_ω network is a 2-layer MLP taking a sinusoidal embedding of time t and outputting a kill rate at time t. `SiLU` activation was used for Q_θ and $\widehat{Q}_{\widehat{\theta}}$; `Leaky-ReLU` for K_ω. All networks used 16-dimensional time embeddings. The ADAM optimizer with gradient clipping was used, with initial learning rates of 0.0001 for all networks (needs to be tuned according to the dataset).

Algorithm S2 SDE Sampling Procedure

Input: drift f, diffusion coefficient ε, killing rate r‘, initial cells n_0, final cells n_T

procedure F-SDE($Q_\theta, \widehat{Q}_{\widehat{\theta}}, K_\omega$)
  sample $\overrightarrow{Z}_0 \sim \rho_0$
  $A_0 \leftarrow 1$
  for step i=1 to T **do**
    $\overrightarrow{Z}_i = \overrightarrow{Z}_{i-1} + \Delta\overrightarrow{Z}_i$
    if $A_{i-1} = 1$ **then**
      $D \sim$ Bernoulli$(1 - k‘(i)\Delta t)$
      $A_{i+1} \leftarrow D$
    end if
  end for
end procedure
procedure B-SDE($Q_\theta, \widehat{Q}_{\widehat{\theta}}, K_\omega$)
  sample $\overleftarrow{Z}_0 \sim \rho_T$
  $A_T \sim$ Bernoulli$(\min(1, \frac{n_T}{n_0}))$
  for step i=T-1 to 0 **do**
    $\overleftarrow{Z}_i = \overleftarrow{Z}_{i-1} - \Delta\overleftarrow{Z}_i$
    if $A_{i+1} = 0$ **then**
      $D \sim$ Bernoulli$(k‘(i)\Delta t)$
      $A_i \leftarrow D$
    end if
  end for
end procedure
Outputs:
  $(Z_t, A_t)_t$

uDSB training is performed within the latent space from the pre-trained VAE. The uDSB training used a batch size of 512 over E_2 iterations, each iteration comprising E_3 epochs of forward and backward training. The uDSB model was trained using the Iterative Proportional Fitting algorithm (IPF), which iteratively solves the schrödinger bridge problem through forward and backward SDEs(Fortet, 1940; Kullback, 1968; Rüschendorf, 1995). During each epoch, the forward and backward drifts, as well as the VAE parameters, are updated (Algorithm S1, Steps 6-20). Specifically, in each epoch, 10 SDEs were sampled to optimize forward and backward drifts each. The process is described below:

1. Forward optimization: This step minimizes the KL divergence with a fixed terminal condition (e.g., $\overleftarrow{Z}_t \sim p_T$). A backward SDE is sampled (Eq. 9b), and the divergence loss is calculated between the SDE predicted by the forward drift and the sampled backward SDE. The VAE params are optimized concurrently using the L_{joint} loss (Eq. 13, Algorithm S1, Steps 9-14))

2. Backward optimization: This step minimizes the KL divergence with a fixed initial condition (e.g., $\overrightarrow{Z}_t \sim p_0$). A forward SDE is sampled (Eq. 9a) and the divergence loss is calculated between the SDE predicted by the backward drift and the sampled forward SDE (Algorithm S1, Steps 15-18)).

The number of discretization steps was set to 100 for the inteval [0,T], so Δt=0.01.

For trajectory inference, gene expression profiles at $t = 0$ are projected into the VAE latent space. Forward SDE sampling (Eq. 9a) with the learned drift Q_θ generates continuous latent variables, decoded back into the gene expression space to reconstruct cellular trajectories.

Prediction performance on held-out timepoints was evaluated by averaging the 2-Wasserstein distance between predicted and ground-truth gene expression profiles,

computed over five forward trajectory samples. The `OTT` library (Cuturi et al., 2022) was used to compute distances.

We performed all training and benchmakring on Linux Ubuntu machine with 256 BG RAM and NVIDIA RTX A6000 GPU with 48 GB RAM. We have reported the runtime and scalability for different training sizes (#cells) on the zebrafish dataset Supplementary Figure S7. The code has also been tested on Linux Ubuntu machine with only CPU.

S2 Baseline Methods

We compare ARTEMIS's performance with the following baseline methods:

- PRESCIENT (Yeo et al., 2021): PRESCIENT (Potential eneRgy undErlying Single Cell gradIENTs) is a generative modeling framework designed to learn differentiation landscapes from time-series scRNA-seq data. It models how cells evolve stochastically and in physical time, using a diffusion-based approach to recover a global potential function. To handle large scRNA-seq datasets, PRESCIENT models are fit on PCA projections of scaled gene expression data. The potential function is parameterized by a neural network, to allow flexible and complex landscape modeling. PRESCIENT allows for using prior knowledge of cell growth in the modeling. However, such information is not always available and we included evaluations with growth rates information when available. We used Python codes on Github (`https://github.com/gifford-lab/prescient-analysis`) to run PRESCIENT.
- MIOFlow (Huguet et al., 2022): MIOFlow (Manifold Interpolating Optimal-Transport Flow) is a computational method for modeling stochastic, continuous population dynamics from snapshots of time-series data. It combines dynamic models, manifold learning, and optimal transport techniques to interpolate between static population snapshots. Using neural ordinary differential equations (Neural ODEs) and a geodesic autoencoder (GAE), MIOFlow ensures the flow aligns with the data's manifold geometry. By operating in the autoencoder's and penalizing transport with Wasserstein distance, complex diffusion processes in cellular dynamics. We use Python codes on GitHub (`https://github.com/KrishnaswamyLab/MIOFlow`) to run MIOFlow
- scNODE (Zhang et al., 2024): scNODE (single-cell Neural Ordinary Differential Equation) is a deep learning model that predicts and simulates single-cell gene expression at unobserved timepoints in temporal scRNA-seq data. It combines a variational autoencoder (VAE) to encode gene expression into a low-dimensional latent space with neural ordinary differential equations (ODEs) to model the temporal evolution of cells within this space. A dynamic regularization term aligns the latent dynamics with temporal data, reducing information loss between discrete timepoints and improving predictions. We use the Python codes on Github (`https://github.com/rsinghlab/scNODE`) to run scNODE.
- uDSB (Pariset et al., 2023): Unbalanced Diffusion Schrödinger Bridge (UDSB) is an extension of the Diffusion Schrödinger Bridge (DSB) framework that allows for modeling the temporal evolution of populations with changing mass over time. Unlike traditional DSBs, which assume conservation of mass and work with probability measures, UDSBs can handle marginals with arbitrary finite mass. uDSBs achieve this by incorporating stochastic differential equations with killing and birth terms, and by deriving their time reversals. We use the Python codes on Github (`https://github.com/matteopariset/unbalanced_sb`) to run scNODE.

S2 Hyperparameter Tuning

To benchmark methods evaluated in this paper, we selected parameters based on the average 2-Wasserstein distance using 3-fold cross validation. Here, we split the cells in each training timepoint into 3 sets, and perform cross-validation such that 2 sets are used

for training, and the third for testing. To search for optimal hyperparameters, we used *wandb* (Biewald, 2020).

For baseline uDSB, we first used PCA to project the gene expression to 50-dimensional space. For the networks Q_θ and $\widehat{Q}_{\widehat{\theta}}$, the `x_encoder` was 3-layer MLP with 300-dimension hidden layers, the `t_encoder` was 3-layer MLP with 32 dimension hidden layers, with takes 16-dimensional sinusoidal embedding of time t, and, the `decoder` was a 3-layer MLP decoder with 300 dimension hidden layers accepts concatenation of outputs from `x_encoder` and `t_encoder`. The K_ω network includes a 5-layer MLP with 64-dimension hidden layers. As activation functions, the `SiLU` for Q_θ and $\widehat{Q}_{\widehat{\theta}}$, and, `Leaky-ReLU` for K_ω networks. The ADAM optimizer wth gradient clipping was used, and initial learning rates for Q_θ and $\widehat{Q}_{\widehat{\theta}}$ were set to 0.001, and 0.01 for K_ω. A batch size of 512 was used, and total training was conducted over 10 iterations, where each iteration included 10 epochs of forward and 10 epochs of backward training.

For PRESCIENT, we searched over the following hyperparameter spaces: latent dimension $\in$ {10,50}, number of hidden layers $\in$ {1,2,3}, sd $\in$ [0.0,1.0], tau $\in$ [0.0,0.1], gradient clipping $\in$ [0.0,1.0]. The remaining hyperparameters were set to default. We estimated the growth rates for the EMT (Cook and Vanderhyden, 2020) and pancreatic (Veres et al., 2019) datasets using the mean of z-scores annotated to birth (KEGG_CELL_CYCLE) and death ()KEGG_APOPTOSIS) as suggested in (Yeo et al., 2021).

For MIOFlow, we searched over the following hyperparameter spaces: gae embedded dim $\in$ {10,50}, layers $\in$ {[50,50],[16,32,16]}, $\lambda \in$ [1,40,100]. The remaining hyperparameters were set to default.

For scNODE, we searched over the following hyperparameter spaces: latent dimension (d) $\in$ {10,50}, encoder network size $\in$ {None, [d], [d,d]}, decoder network size $\in$ {None, [d], [d,d]}, drift network size $\in$ {None, [d], [d,d]}.

For ARTEMIS, we searched over the following hyperparameter spaces: latent dimension $\in$ {10,50}, vae encoder hidden dimension $\in$ {[512,256], [256,128]}, vae decoder hidden dimension $\in$ {[512,256], [256,128]}, vae pre-train epochs (E_1) $\in$ {50,100}, number of iterations (E_2) $\in$ {2,4,6,8,10}, all learning rates $\in$ {0.001,0.0001}, vae batch size $\in$ {32, 64}, SDE sampling batch size $\in$ {256,512}.

S4 Investigate ARTEMIS hyperparameters

We next investigate the hyperparameters tuned in ARTEMIS and their effects on prediction performance at unmeasured timepoints. Using the three datasets (Veres et al., 2019; Farrell et al., 2018; Cook and Vanderhyden, 2020), we evaluate its performance on the previously defined task on held out timepoints. We hope this analysis provides heuristic guidance to users to choose hyperparameters for training ARTEMIS. We vary the following hyperparameters:

1. base drift f from {0,2,4,8,10,50,100}: Supplementary Figure S5a. compares ARTEMIS's performance across different values of the base drift f. Lower values lead to better prediction performance, whereas higher values can dominate the learned forward and backward drifts, resulting in trajectories primarily driven by the base drift rather than the learned dynamics.
2. Number of discretization steps from {12,50,75,100}: As shown in Supplementary Figure S5b., the prediction performance remains largely consistent across different numbers of discretization steps. Therefore, users may select the number of steps based on the desired level of trajectory resolution.
3. VAE latent dimension d from [10,25,50,75,100,125,175,200]: Supplementary Figure S5c. compares the VAE latent dimensions. We find that a lower latent dimension (e.g., 10) is sufficient for simpler datasets. However, for more complex datasets with

multiple cell types, we recommend using larger latent dimensions (>50) to better capture variability.

4. SDE sampling batch size from {32,64,128,256,512}" Supplementary Figure S5d. shows that a higher sampling size improves performance as it effectively penalizes more samples during training within a fixed number of epochs. However, a larger sampling size could cause computational overhead, thus, users can make a reasonable choice based on a tradeoff between accuracy and computational costs.
5. Effect of using L_{joint} loss terms, i.e., including either, both, or none of the terms: The results in Supplementary Figure S5e. indicate that including both losses enables joint optimization of the VAE and uDSB components, leading to better performance compared to using either loss individually or omitting them entirely.

S5 Identify drift-genes

To bridge the latent forward drift dynamics with gene expression changes, we map the learned latent drift values to the gene expression space. Let $\overrightarrow{z}_{t,i} \in \mathcal{R}^d$ be a latent variable generated using the forward SDE (Eq. 9a) for a cell i at time t. Let $\widehat{x}_{t,i} \in \mathcal{R}^g$ be the reconstructed gene expression for the cell i using the decoder (p_ϕ). To map the forward drift at t to the gene expression space, we multiply the output of $Q^\theta(\overrightarrow{z}_{t,i}, t) \in \mathcal{R}^d$ with the jacobian of p_ϕ to compute the jacobian vector product (JVP) (RIRSCH and Smale, 1974).

The jacobian $J \in \mathcal{R}^{g \times d}$ of p_ϕ is given by:

$$J_i = \begin{bmatrix} \frac{\partial \widehat{x}_{t,i,(1)}}{\partial \overrightarrow{z}_{t,i,(1)}} & \frac{\partial \widehat{x}_{t,i,(1)}}{\partial \overrightarrow{z}_{t,i,(2)}} & \cdot\cdot & \frac{\partial \widehat{x}_{t,i,(1)}}{\partial \overrightarrow{z}_{t,i,(d)}} \\ \cdot & \cdot & \cdot & \cdot \\ \cdot & \cdot & \cdot & \cdot \\ \frac{\partial \widehat{x}_{t,i,(g)}}{\partial \overrightarrow{z}_{t,i,(1)}} & \frac{\partial \widehat{x}_{t,i,(g)}}{\partial \overrightarrow{z}_{t,i,(2)}} & \cdot\cdot & \frac{\partial \widehat{x}_{t,i,(g)}}{\partial \overrightarrow{z}_{t,i,(d)}} \end{bmatrix}, \tag{1}$$

Then, the drift scores for cell i can be calculated as:

$$\text{drift\_scores}_i = J_i Q_\theta(\overrightarrow{z}_{t,i}, t), \tag{2}$$

where gene_drift_scores$_i \in \mathcal{R}^g$. This is then averaged over all cells at time t to get an average score for each gene:

$$\text{drift\_scores} = \frac{\sum_{i=1}^{n_t} J_i Q^\theta(\overrightarrow{z}_{t,i}, t)}{n}, \tag{3}$$

where drift_scores $\in \mathcal{R}^g$ and drift_scores$_j$ gives a gene-drift-score for gene j. We used the `jax.jvp()` function from JAX library for computing forward-mode Jacobian-vector product.

Significance testing of drift-genes

To evaluate the significance of the gene-drift scores, we randomized the positions of cells in the latent space and calculated their forward drifts. The drift values from the randomized cells, combined with the decoded gene expression of the original unshuffled cells, were used to establish a null distribution for gene-drift scores. A two-sided t-test was then performed to compare the observed gene-drift scores against the null distribution. Most drift genes identified by ARTEMIS were found to be significant, with p-values < 0.05.

Drift gene bias toward highly expressed genes

To assess potential bias, we computed the Spearman correlation between drift scores and average gene expression across three datasets (Supplementary Figure S6a.). While

some timepoints showed high correlation (> 0.5), several had lower correlations (< 0.5). We also compared the top 20 drift and highly expressed genes, finding low overlap (< 10 genes) at several timepoints and high overlap (> 10 genes) at some others (Supplementary Figure S6b.). In cases of high overlap, we found the genes to be biologically relevant, including progenitor genes at t=0,1 and SC-β/SC-EC branch-associated genes at t=7 in the pancreatic dataset (Veres et al., 2019), as well as key developmental regulators in zebrafish (Farrell et al., 2018). At timepoints with low overlap (< 10 genes), drift genes had significantly lower expression than highly expressed genes. These findings show that while some drift genes are highly expressed, many are not driven by high expression.

S6 Perturbation analysis

For the EMT dataset (Cook and Vanderhyden, 2020), *in silico* perturbations were introduced by modifying the scaled normalized expression of target genes to z-score values: less than 0 for underexpression (knockdowns) and greater than 0 for overexpression. The resulting perturbed gene expression profile was then input to the trained ARTEMIS model, where it is was projected to the latent space to generate a forward SDE up to time T. Perturbations were introduced with magnitudes of -25,-20,-15,-10,10,15,20,25.

We conducted 10 trials, where 2,000 cells sampled from the timepoint of perturbation introduction were used to predict cellular trajectory up to time T using the pre-trained ARTEMIS model. These simulations allowed us to examine the effects of perturbations within the latent space.

We trained a multilayer perceptron (MLP) classifier using the Python library scikit-learn (Pedregosa et al., 2011) to predict timepoints based on latent cell representations. For a given trajectory resulting from perturbations introduced at time t, the predicted trajectory in the latent space was classified using the pre-trained MLP classifier.

Then, the number of predicted cells classified into each timepoint were compared between perturbed and unperturbed trajectories by performing a two-sided t-test test. We used this analysis to identify if the introduced perturbations for the drift-genes could alter/reverse the epithelial-to-mesenchymal transition by generating more cells corresponding to earlier or later timepoints (Figure 5, Supplementary Figure S4).

S7 Performance on sparse datasets

We further evaluated ARTEMIS on a mouse hematopoiesis dataset with lineage tracing information spanning three timepoints, comprising 49,302 cells (Weinreb et al., 2020). To assess its predictive performance, we withheld the second time point. ARTEMIS demonstrated superior accuracy in reconstructing the held out time point (see Supplementary Table S4), indicating its robustness even in sparse settings. However, a limited number of time points may reduce the resolution of inferred trajectories, particularly when key transitions occur between unobserved intervals.

Evaluating clonal fate prediction

We further evaluated ARTEMIS on lineage tracing data to assess its ability to predict clonal fate within neutrophil and monocyte lineages. Here, we trained ARTEMIS on all timepoints and initialized the trained model with cells from $t = 0$ and having clonal information across three timepoints to predict forward trajectories. Using the trained model, the inferred forward cell drift captured the differentiation lineages (see Supplementary Figure S9a.), recovered relative cell population changes, and predicted cell statuses (see Supplementary Figure S9b.).

For clonal bias analysis, cells at the final timepoint were classified as neutrophil, monocyte, or others using a nearest neighbor classifier trained on the groundtruth gene expression. Following the approach in [1], we computed clonal fate probability for each cell at $t = 0$as the fraction of its clonal relatives that became neutrophils or monocytes as

ground truth. For ARTEMIS predicted trajectories, this was approximated as the number of neutrophils divided by the total number of monocytes and neutrophils within each cell's trajectory (see Supplementary Figure S9c.). ARTEMIS recovered clonal bias well for the neutrophil lineage and weakly for the monocyte lineage.

Data preprocessing

The mouse hematopoiesis dataset is available at `https://github.com/AllonKleinLab/paper-data`. We used cells from all three timepoints with lineage tracing information, log-normalized the training and held out time points separately, selected the top 2,000 highly variable genes, and removed cell cycle genes based on the training set, following the recommendations in (Jiang and Wan, 2024). We used the same strategy as in Supplementary Note S1 for evaluating prediction performance.

S8 Effect of cell population modeling in ARTEMIS performance

ARTEMIS models relative cell population changes using cell counts as ground truth rather than relying on prior knowledge, such as proliferation or apoptosis-associated genes. To assess the effect of cell population modeling, we trained a variant of ARTEMIS without the neural network for kill rate inference and compared its performance across three datasets for training and held out timepoints (Supplementary Figure S8). We also show a comparison with the PRESCIENT model trained with growth rates inferred from prior knowledge. Results show that ARTEMIS performs similarly with and without explicit cell population modeling, as the kill rate network is trained independently and can be excluded, allowing uDSBs to function as diffusion Schrödinger bridges (DSBs). However, incorporating cell population modeling enables ARTEMIS to infer cell population changes.

Supplementary Figures

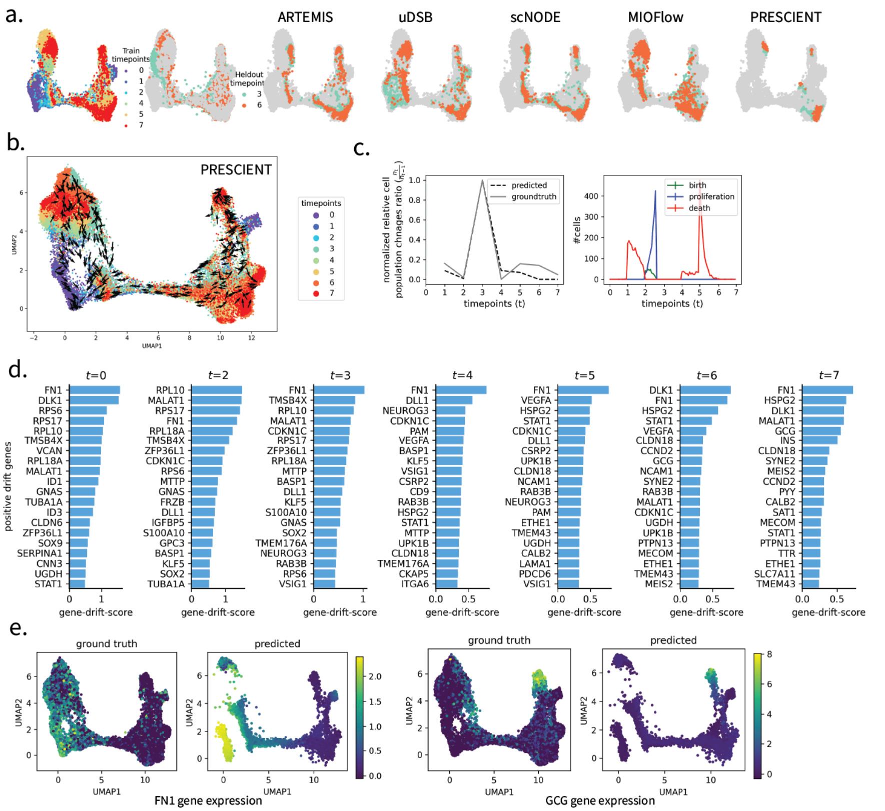

Supplementary Figure S1. Application to β-cell differentiation in human pancreas. a.) Benchmarking ARTEMIS against state-of-the-art methods for predicting gene expression at held-out timepoints (3,6) using pancreatic data. b.) Cell drift inferred by Prescient (w.o. growth rates). c.) Left: Comparison of normalized ratios of relative cell population changes between ground truth and ARTEMIS-predicted cell statuses as live, Right: Number of cells predicted as born, proliferated, and died throughout the trajectory. An increase in cell births and proliferation was observed at $t = 2$ and $t = 3$, coinciding with an increase in relative cell population in the ground truth data. Conversely, a significant number of cells were predicted to die between $t = 1$ to $t = 2$ and $t = 4$ to $t = 6$, aligning with the observed decline in relative cell population. d.)Drift genes identified for zebrafish dataset for remaining ten timepoints. e.) Ground truth vs. predicted gene expression of drift genes FN1 (identified across all timepoints) and GCG (identified at t=6,7).

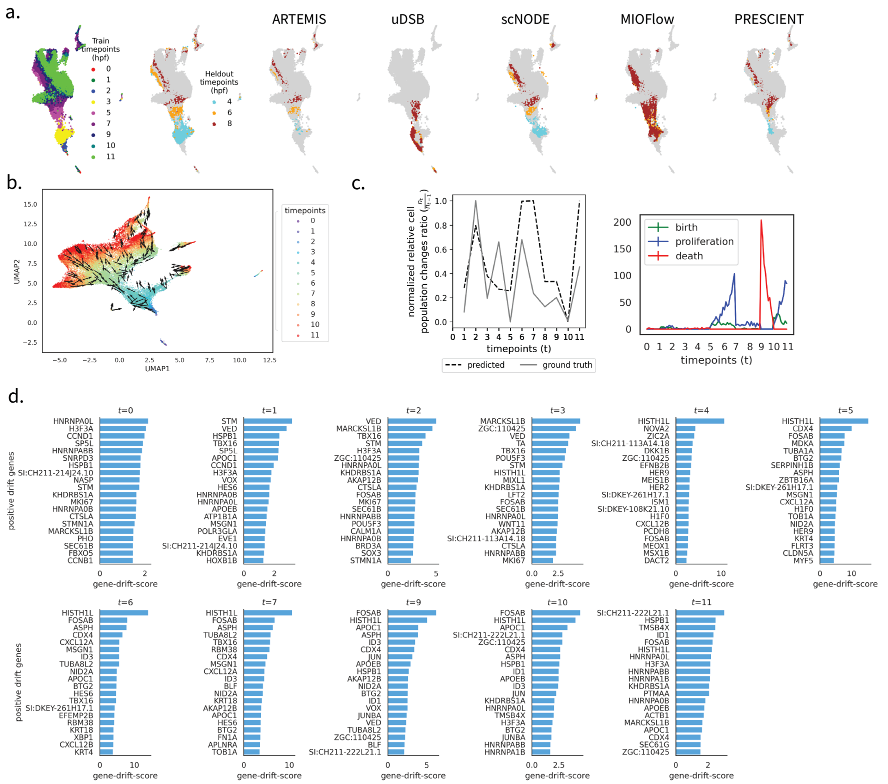

Supplementary Figure S2. Application to Zebrafish embryogeneis data. a.) Benchmarking ARTEMIS against state-of-the-art methods for predicting gene expression at held-out timepoints (4, 6, 8) using zebrafish data. b.) Cell drift inferred by PRESCIENT. c.) Left: Comparison of normalized ratios of relative cell population changes between ground truth and ARTEMIS-predicted cell statuses as live, Right: Number of cells predicted as born, proliferated, and died throughout the trajectory. An increase in cell births and proliferation was observed between $t = 4$ to $t = 7$ and $t = 10$ to $t = 11$, coinciding with an increase in relative cell population in the ground truth data. Conversely, a significant number of cells were predicted to die between $t = 9$ to $t = 10$, aligning with the observed decline in relative cell population. d.) Drift genes identified for zebrafish dataset for remaining ten timepoints.

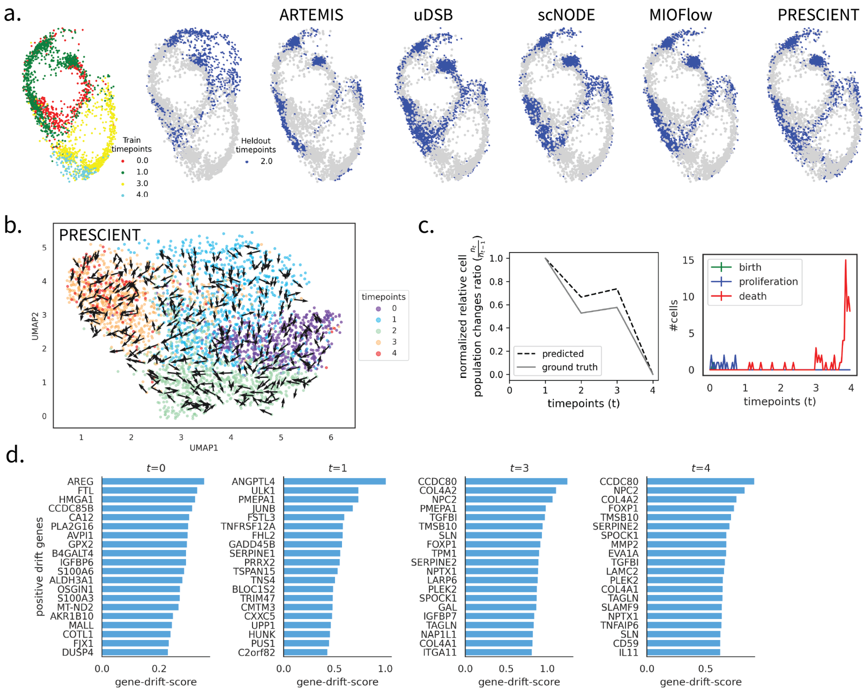

Supplementary Figure S3. Application to A549 lung cancer cells treated with *TFGB1* to induce EMT. a.) Benchmarking ARTEMIS against state-of-the-art methods for predicting gene expression at held-out timepoint (2) using EMT data. b.) Cell drift inferred by PRESCIENT. c) Left: Comparison of normalized ratios of relative cell population changes between ground truth and ARTEMIS-predicted cell statuses as live, Right: Number of cells predicted as born, proliferated, and died throughout the trajectory. Shorter intervals of predicted cell proliferation are observed earlier in the trajectory, potentially reflecting the higher cell numbers in the ground truth data at these early timepoints. Cell death events are distributed across several shorter intervals throughout the trajectory. A significant spike in predicted cell death is observed towards the end of the trajectory, coinciding with a substantial reduction in the relative cell population in the ground truth data. d.) Drift genes identified for emt dataset for remaining four timepoints.

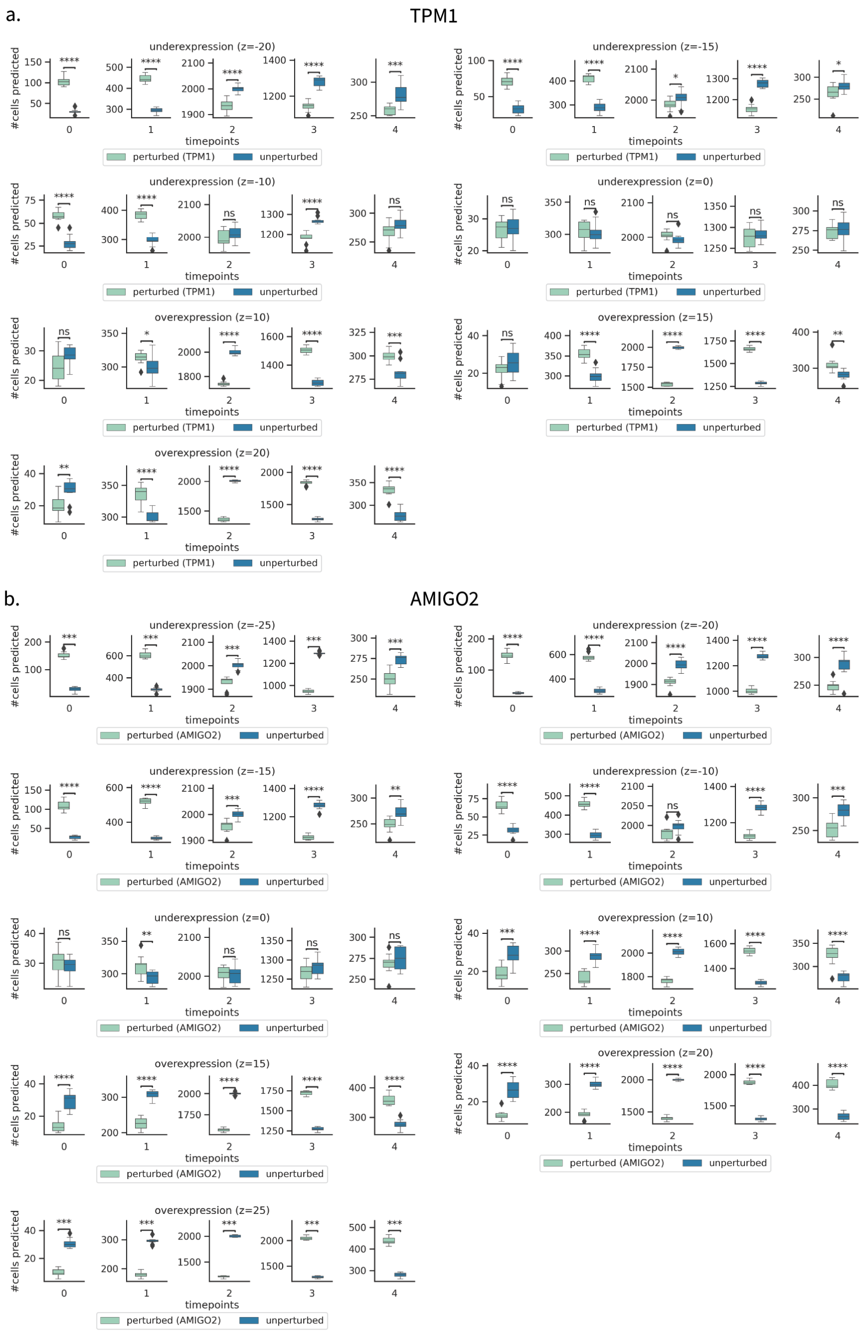

Supplementary Figure S4. Perturbation results for different levels of over and underexpression of genes (a) *TPM1* and (b) *AMIGO2*: (-20,-15,-15,-10,0,10,15,20) and additionally (-25,25) for *AMIGO2*. Cells are assigned to specific timepoints by an MLP classifier, and the number of cells generated from perturbed and unperturbed trajectories were compared using a two-sided t-test at $p < 0.05$.

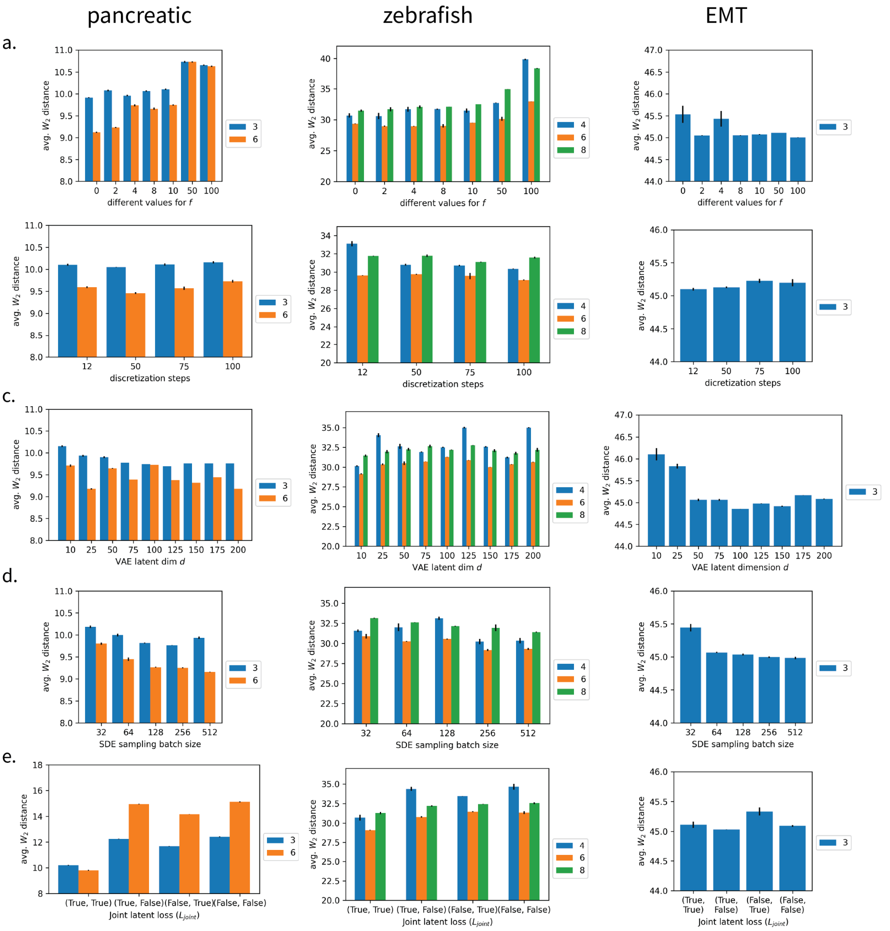

Supplementary Figure S5. Evaluating different hyperparameter sets and training configurations for ARTEMIS. The different hyperparameters/configurations are: a.) base drift f, b.) discretization steps, c.) VAE latent dimension$d$, d.) SDE sampling batch size, e.) effect of using $L_{joint} = W_2(Z_{\varphi,t}, \overrightarrow{Z}_t) + W_2(X_t, p_\phi(Z_{\varphi,t}))$ loss terms. The x-axis $(\cdot,\cdot)$ indicates when either of the W_2 losses is used.

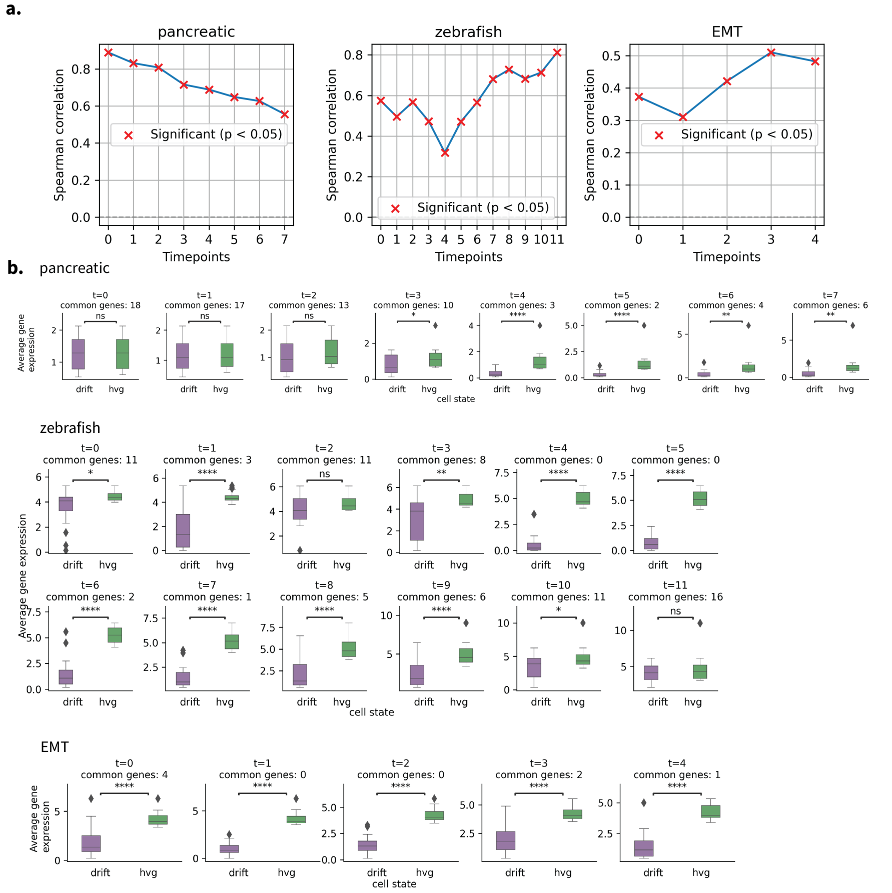

Supplementary Figure S6. a.) Spearman correlation between drift gene scores and average gene expression to assess potential bias toward highly expressed genes. b.) Comparison of expression levels between the top 20 drift genes and the top 20 highly expressed genes at each timepoint, including the number of common or overlapping genes between the two sets.

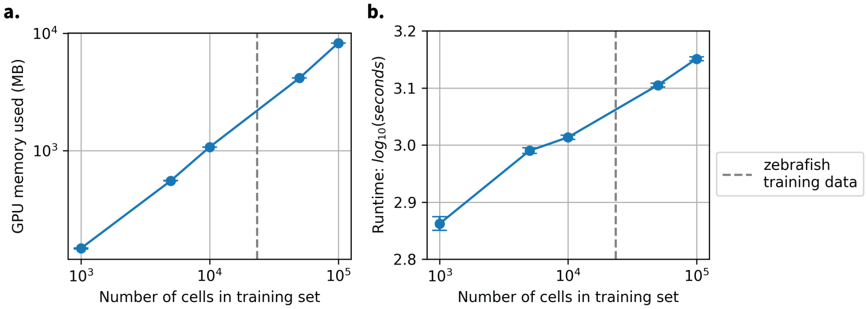

Supplementary Figure S7. a.) memory and b.) runtime estimates for increasing training set sizes tested on the NVIDIA RTX A6000 GPU using the zebrafish dataset. The hyperparameters used in training are: base drift f=2, VAE latent dim=10, SDE sampling batch size=256, discretization steps=100, VAE pre-train epochs=50.

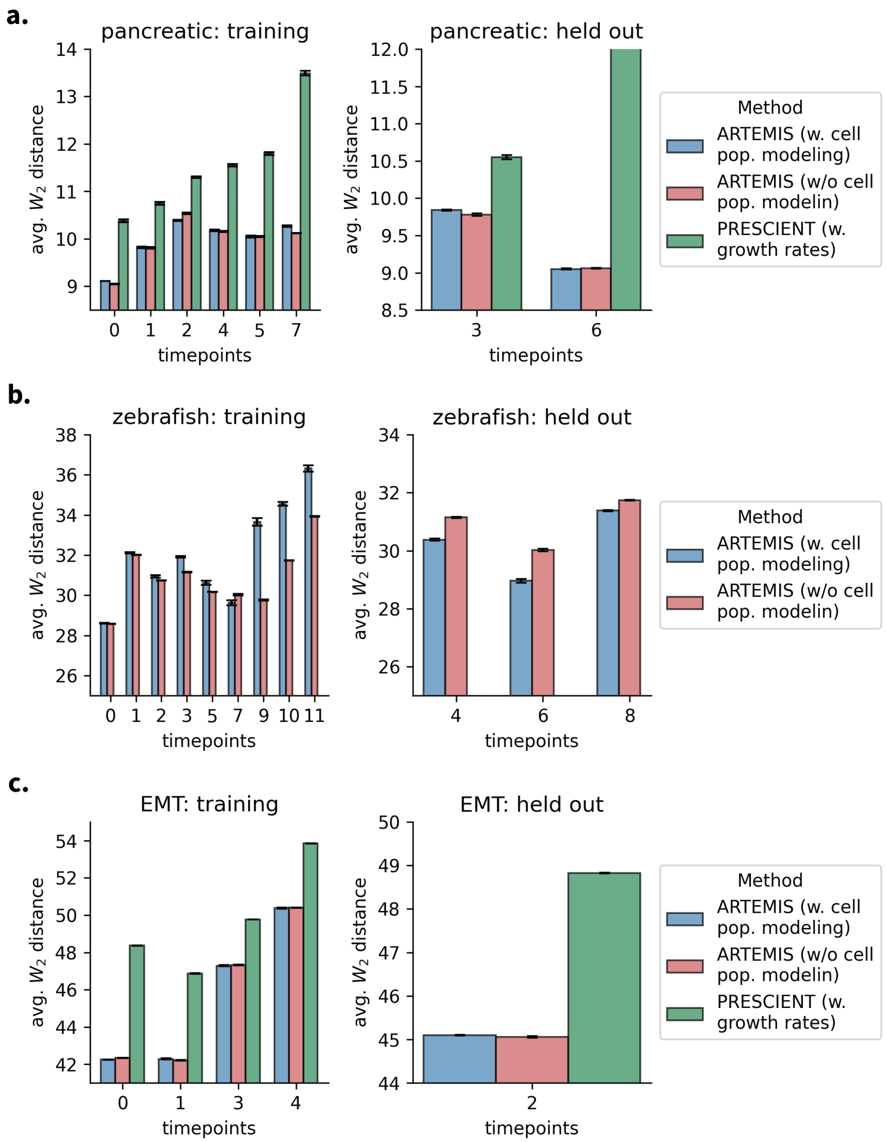

Supplementary Figure S8. a.- c.) Training and held out performance comparing ARTEMIS trained with and without cell population modeling across three datasets, also compared to Prescient trained with growth rates from prior knowledge.

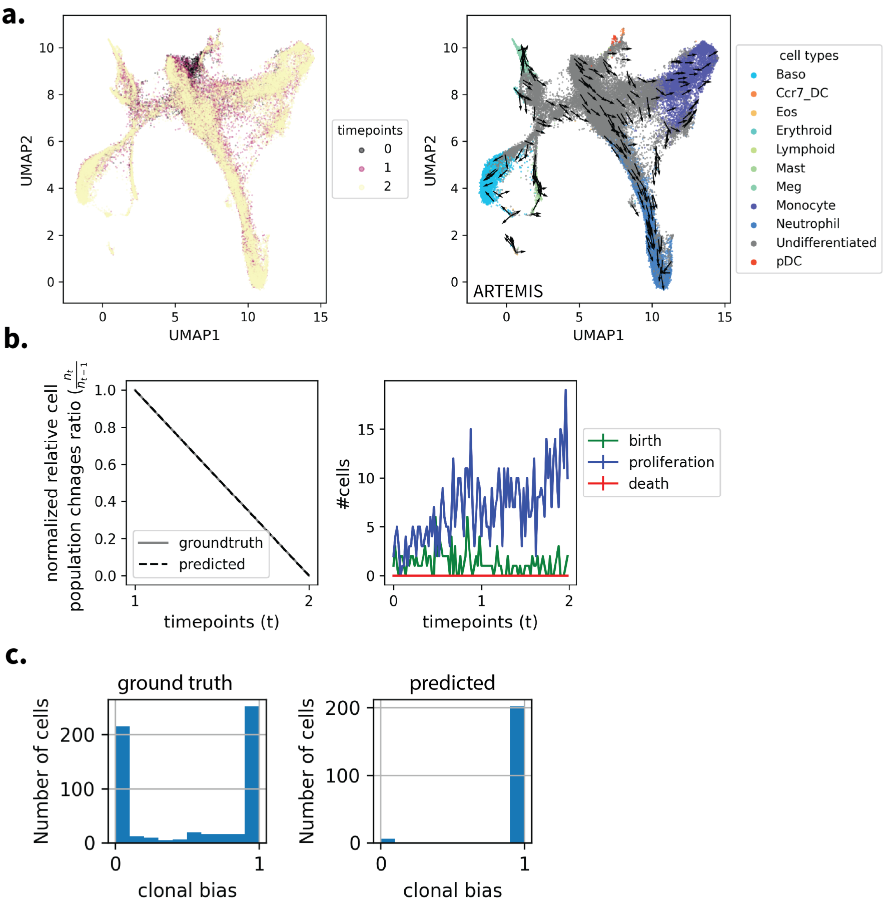

Supplementary Figure S9. Application to mouse hematopoiesis. a.) Left: ground truth data colored by time. Right: Visualization of the drift inferred by ARTEMIS trained on all timepoints. b.) Left: Comparison of normalized ratios of relative cell population changes between ground truth and ARTEMIS-predicted cell statuses as live. Right: Number of cells predicted as born, proliferated, and died across the trajectory. Cell proliferation increases throughout the differentiation landscape as the cell population increases in the ground truth data. The rate of cell proliferation and birth is higher from $t = 0$ to $t = 1$ compared to that between $t = 1$ to $t = 2$, as the increase in cell population from $t = 1$ to $t = 2$ is 2 times compared to that from $t = 0$ to $t = 1$, which was 3.2 times. c.) The distribution of ground truth and predicted clonal fate bias for neutrophil and monocyte lineages is computed as the number of neutrophils divided by the total number of monocytes and neutrophils within each cell's trajectory.

Supplementary Tables

Supplementary Table S1. Wasserstein distance between the predicted and training timepoints for the pancreatic dataset (Veres et al., 2019). Numbers in **bold** indicate best performance.

	t=0	t=1	t=2	t=4	t=5	t=7
**ARTEMIS**	**9.11±0.00**	**9.82±0.02**	**10.39±0.01**	**10.18±0.02**	**10.05±0.02**	**10.27±0.02**
**uDSB**	9.38±0.002	10.81±0.01	11.89±0.01	11.94±0.01	11.94±0.01	11.94±0.001
**scNODE**	9.5±0.001	10.08±0.001	10.54±0.00	10.35±0.001	10.21±0.001	10.29±0.001
**MIOFlow**	12.22±0.01	11.73±0.01	11.73±0.01	11.49±0.01	11.58±0.02	12.12±0.02
**Prescient**	10.38±0.03	10.73±0.02	11.22±0.01	11.29±0.01	11.38±0.01	12.48±0.02
**Prescient (w. growth rates)**	10.38±0.03	10.75±0.03	11.3±0.02	11.55±0.03	11.8±0.03	13.49±0.05

Supplementary Table S2. Wasserstein distance between the predicted and training timepoints for the zebrafish dataset (Farrell et al., 2018). Numbers in **bold** indicate best performance.

	t=0	t=1	t=2	t=3	t=5
**ARTEMIS**	**28.6±0.021**	**32.11±0.03**	**30.94±0.07**	**31.92±0.04**	**30.62±0.11**
**uDSB**	29.27±0.009	34.08±0.05	37.18±0.2	42.5±0.22	44.87±0.18
**scNODE**	29.66±0.002	32.72±0.001	31.75±0.002	32.17±0.002	30.76±0.003
**MIOFlow**	46.42±0.02	43.75±0.02	35.33±0.02	34.68±0.01	33.90±0.02
**Prescient**	61.35±0.2	61.71±0.22	54.47±0.21	53.04±0.2	51.80±0.2

	t=7	t=9	t=10	t=11
**ARTEMIS**	**29.62±0.13**	33.66±0.19	**34.55±0.09**	36.32±0.16
**uDSB**	42.88±0.07	44.96±0.06	45.82±0.08	46.91±0.08
**scNODE**	30.55±0.003	**33.66±0.002**	35.12±0.001	**36.06±0.002**
**MIOFlow**	33.11±0.04	37.22±0.04	39.55±0.04	41.79±0.05
**Prescient**	48.73±0.26	52.99±0.26	56.61±0.25	58.71±0.26

Supplementary Table S3. Wasserstein distance between the predicted and training timepoints for the EMT dataset (Cook and Vanderhyden, 2020). Numbers in **bold** indicate best performance.

	t=0	t=1	t=3	t=4
**ARTEMIS**	**42.25±0.01**	**42.29±0.03**	**47.29±0.03**	**50.38±0.03**
**uDSB**	42.28±0.05	44.18±0.12	49.64±0.12	52.34±0.10
**scNODE**	42.75±0.002	42.70±0.003	49.7±0.01	52.45±0.02
**MIOFlow**	44.98±0.02	42.96±0.01	49.68±0.02	56.08±0.06
**Prescient**	45.31±0.03	44.33±0.05	47.99±0.03	51.60±0.01
**Prescient (w. growth rates)**	48.37±0.02	46.88±0.02	49.77±0.01	53.85±0.01

Supplementary Table S4. Wasserstein distance between the predicted and held-out timepoints for mouse hematopoiesis dataset. Numbers in **bold** indicate best performance.

Timepoints	**train**		**held out**
	t=0	t=2	t=1
**ARTEMIS**	3.69±0.015	4.99±0.019	4.62±0.04
**uDSB**	3.86±0.02	5.7±0.04	6.25±0.05
**scNODE**	3.82±0.0001	**4.94±0.0002**	**4.54±0.0005**
**MIOFlow**	**3.66±0.0002**	5.46±0.011	4.87±0.009
**Prescient**	4.8±0.003	7.2±0.004	5.8±0.003

References

L. Biewald. Experiment tracking with weights and biases, 2020. URL `https://www.wandb.com/`. Software available from wandb.com.

J. Bradbury, R. Frostig, P. Hawkins, M. J. Johnson, C. Leary, D. Maclaurin, G. Necula, A. Paszke, J. VanderPlas, S. Wanderman-Milne, and Q. Zhang. JAX: composable transformations of Python+NumPy programs. 2018. URL `http://github.com/jax-ml/jax`.

D. P. Cook and B. C. Vanderhyden. Context specificity of the emt transcriptional response. *Nature communications*, 11(1):2142, 2020.

M. Cuturi, L. Meng-Papaxanthos, Y. Tian, C. Bunne, G. Davis, and O. Teboul. Optimal transport tools (ott): A jax toolbox for all things wasserstein. *arXiv preprint arXiv:2201.12324*, 2022.

J. A. Farrell, Y. Wang, S. J. Riesenfeld, K. Shekhar, A. Regev, and A. F. Schier. Single-cell reconstruction of developmental trajectories during zebrafish embryogenesis. *Science*, 360(6392):eaar3131, 2018.

R. Fortet. Résolution d'un système d'équations de m. schrödinger. *Journal de Mathématiques Pures et Appliquées*, 19(1-4):83–105, 1940.

G. Huguet, D. S. Magruder, A. Tong, O. Fasina, M. Kuchroo, G. Wolf, and S. Krishnaswamy. Manifold interpolating optimal-transport flows for trajectory inference. *Advances in neural information processing systems*, 35:29705–29718, 2022.

Q. Jiang and L. Wan. A physics-informed neural sde network for learning cellular dynamics from time-series scrna-seq data. *Bioinformatics*, 40(Supplement_2):ii120–ii127, 2024.

S. Kullback. Probability densities with given marginals. *The Annals of Mathematical Statistics*, 39(4):1236–1243, 1968.

M. Pariset, Y.-P. Hsieh, C. Bunne, A. Krause, and V. D. Bortoli. Unbalanced diffusion schrödinger bridge. *ICML Workshop on New Frontiers in Learning, Control, and Dynamical Systems*, abs/2306.09099, 2023. URL `https://api.semanticscholar.org/CorpusID:259165197`.

F. Pedregosa, G. Varoquaux, A. Gramfort, V. Michel, B. Thirion, O. Grisel, M. Blondel, P. Prettenhofer, R. Weiss, V. Dubourg, J. Vanderplas, A. Passos, D. Cournapeau, M. Brucher, M. Perrot, and E. Duchesnay. Scikit-learn: Machine learning in Python. *Journal of Machine Learning Research*, 12:2825–2830, 2011.

M. W. RIRSCH and S. Smale. *Differential equations, dynamical systems, and linear algebra*. ACADEMIC PRESS. INC., 1974.

L. Rüschendorf. Convergence of the iterative proportional fitting procedure. *The Annals of Statistics*, pages 1160–1174, 1995.

A. Veres, A. L. Faust, H. L. Bushnell, E. N. Engquist, J. H.-R. Kenty, G. Harb, Y.-C. Poh, E. Sintov, M. Gürtler, F. W. Pagliuca, et al. Charting cellular identity during human in vitro β-cell differentiation. *Nature*, 569(7756):368–373, 2019.

C. Weinreb, A. Rodriguez-Fraticelli, F. D. Camargo, and A. M. Klein. Lineage tracing on transcriptional landscapes links state to fate during differentiation. *Science*, 367(6479): eaaw3381, 2020.

G. H. T. Yeo, S. D. Saksena, and D. K. Gifford. Generative modeling of single-cell time series with prescient enables prediction of cell trajectories with interventions. *Nature communications*, 12(1):3222, 2021.

J. Zhang, E. Larschan, J. Bigness, and R. Singh. scnode: generative model for temporal single cell transcriptomic data prediction. *Bioinformatics*, 40(Supplement_2):ii146–ii154, 2024.
